# Protective Effect of Aqueous Crude Extract of Neem (*Azadirachta indica*) Leaves on *Plasmodium berghei*-Induced Renal Damage in Mice

**DOI:** 10.1155/2015/961205

**Published:** 2015-08-25

**Authors:** Voravuth Somsak, Sukanya Chachiyo, Ubonwan Jaihan, Somrudee Nakinchat

**Affiliations:** Department of Clinical Chemistry, Faculty of Medical Technology, Western University, Kanchanaburi 71170, Thailand

## Abstract

Malaria is a major public health problem in the world because it can cause of death in patients. Malaria-associated renal injury is associated with 45% of mortality in adult patients hospitalized with severe form of the disease. Therefore, new plant extracts to protect against renal injury induced by malaria infection are urgently needed. In this study, we investigated the protective effect of aqueous crude extract of *Azadirachta indica* (neem) leaves on renal injury induced by *Plasmodium berghei* ANKA infection in mice. ICR mice were injected intraperitoneally with 1 × 10^7^ parasitized erythrocytes of PbANKA, and neem extracts (500, 1,000, and 2,000 mg/kg) were given orally for 4 consecutive days. Plasma blood urea nitrogen (BUN) and creatinine levels were subsequently measured. Malaria-induced renal injury was evidenced as marked increases of BUN and creatinine levels. However, the oral administration of neem leaf extract to PbANKA infected mice for 4 days brought back BUN and creatinine levels to near normalcy, and the highest activity was observed at doses of 1,000 and 2,000 mg/kg. Additionally, no toxic effects were found in normal mice treated with this extract. Hence, neem leaf extract can be considered a potential candidate for protection against renal injury induced by malaria.

## 1. Introduction

Malaria caused by* Plasmodium* parasites is a major public health problem in tropical zones including Africa, South and Central America, and Asia, where it is most prevalent, with estimates of 216 million cases and 655,000 deaths. More than 85% of malaria cases and 90% of malaria deaths occur in sub-Saharan Africa, mainly in young children [1]. Causes of death in malaria have been reported including cerebral malaria, acute hemolysis and severe anemia, and vital organ damage and failure [2]. For organ damage, malaria-associated renal injury occurs in between 1 and 4% of hospitalized patients with a mortality that can reach up to 45% [3]. The pathogenesis of malaria-associated renal injury is multifactorial and not well characterized, but several hypotheses suggest involvement of cytoadherence of parasitized erythrocytes. Recent studies suggest that proinflammatory response, oxidative injury, and apoptosis probably explain part of this injury [4, 5]. During malaria infection, it causes generation of reactive oxygen species (ROS), such as superoxide anion and hydroxyl radical which deplete glutathione levels and inhibit the activity of antioxidant enzymes, especially in renal tissues [6, 7]. Therefore, oxidative stress induced by malaria infection may produce cellular injury and necrosis via several mechanisms including peroxidation of membrane lipids, protein denaturation, and DNA damage [8].

In this respect, medicinal plants are potential targets for research and development of alternative drugs. In particular,* Azadirachta indica* (neem) is one of the most promising medicinal plants, having several biological activities, especially as antioxidant, anti-inflammatory, antibacterial, antifungal, and antiulcer ones [9–12]. Several studies have been undertaken on the protective effects of neem [13–15]. It has been described that aqueous crude extract of neem leaves showed significant hypoglycemic, hypolipemic, hepatoprotective, and hypertensive activities [16–19]. Moreover, protective effect on diabetic nephropathy in rats of neem leaf extract has also been reported [20]. However, the protective effect of neem extract on renal damage induced by malaria infection has not yet been reported. Hence, the aim of the present study was to evaluate the possible protective effect of aqueous crude extract of neem leaves on* P. berghei*-induced renal damage in mice.

## 2. Materials and Methods

### 2.1. Crude Extract Preparation

The neem leaves were collected from Kanchanaburi province, Thailand. The plants were identified in the Faculty of Pharmacy, Payap University. The leaves were cleaned and dried using hot air oven at 55°C for 6 h and then they were powdered. The powder was used for the preparation of aqueous crude extract according to the procedure previously described with some modification [10]. The dried powders of neem leaves were boiled with distilled water (plant : water = 1 : 20, w/v) for 6 h and then filtered. The filtrate was evaporated to dryness in a vacuum evaporator to yield the aqueous crude extract of neem leaf. Before experiment, the neem leaf extract was dissolved in 20% Tween-80 at the doses of 500, 1,000, and 2,000 mg/kg.

### 2.2. Mice

Female ICR mice obtained from the National Laboratory Animal Center, Mahidol University, Thailand, 4–6 weeks old, weighing 25 to 30 g were used for the study. They were given clean tap water and pelleted diet* ad libitum*. All mouse procedures were approved by the Ethical Committee on Animal Experimentation, Faculty of Medical Technology, Western University, Thailand.

### 2.3. Parasite Strain and Infection of Animals

*Plasmodium berghei* ANKA (PbANKA) obtained from Dr. Chairat Uthaipibull from BIOTEC, NSTDA, Thailand, was used. Naïve ICR mice were inoculated with 1 × 10^7^ parasitized erythrocytes of PbANKA by intraperitoneal (IP) injection. Parasitemia was daily monitored by microscopy of Giemsa stained thin blood smear. Additionally, renal markers including BUN and creatinine levels were also measured.

### 2.4. Measurements of Renal Markers

For determination of renal damage, BUN and creatinine levels were used as markers in this study. Blood was collected from tail vein of mice into heparinized hematocrit tubes. Centrifugation was then performed with 10,000 g for 10 min. Next, plasma was collected into a new 1.5-mL tube for measurement of BUN and creatinine using commercially available diagnostic kits (BioSystems S. A. Costa Brava, Barcelona, Spain) according to the manufacturer's instructions.

### 2.5. Efficacy Test* In Vivo*

The standard 4-day suppressive test against PbANKA infection in mice was employed [21]. Groups of naïve ICR mice (5 mice of each) were inoculated with 1 × 10^7^ parasitized erythrocytes of PbANKA by IP injection. They were subsequently treated with 500, 1,000, and 2,000 mg/kg of neem leaf extract orally by gavage twice a day for 4 consecutive days. Normal mice treated with or without 2,000 mg/kg of extract were used as healthy controls, while PbANKA infected mice treated with 20% Tween-80 were used as disease controls. On day 5 of experiment, blood was collected for measurement of BUN and creatinine levels.

### 2.6. Statistical Analysis

The one-way ANOVA was used to analyze and compare the results at a 95% confidence level. Values of *P* < 0.05 were considered significant. Results were expressed as mean ± standard error of mean (SEM).

## 3. Results

### 3.1. Renal Damage Induced by* Plasmodium berghei* Infection in Mice

**Figure 1:**
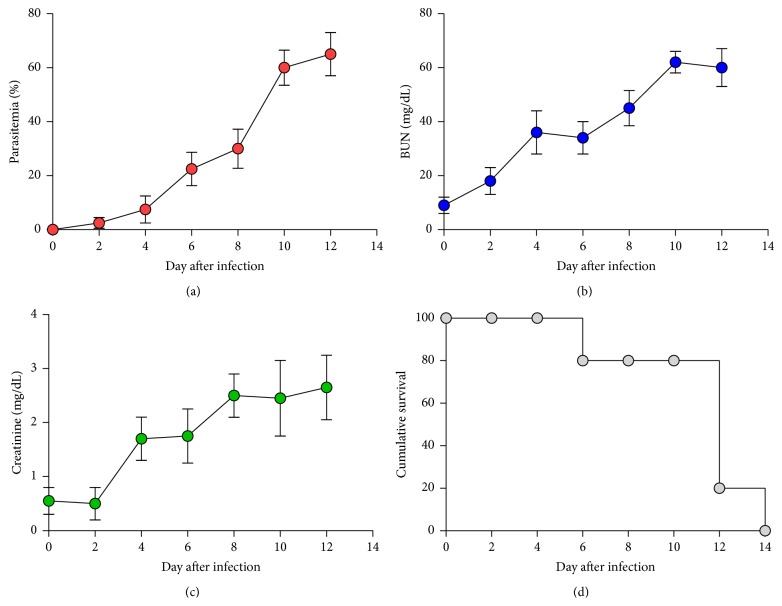
Malaria-associated renal injury induced by* Plasmodium berghei* infection in mice. Groups of ICR mice (5 mice of each) were infected with 1 × 10^7^ parasitized erythrocytes of PbANKA by IP injection. (a) Parasitemia, (b) BUN, and (c) creatinine levels were daily measured. (d) Cumulative survival of infected mice was also observed. Results were expressed as mean ± SEM.

ICR mice were infected with 1 × 10^7^ parasitized erythrocytes of PbANKA by IP injection; parasitemia and renal markers were then daily monitored. It was found that PbANKA was firstly detectable on day 2 after infection with a parasitemia less than 1% and reached >60% on day 12 (Figure 1(a)). Moreover, during blood stage propagation of PbANKA in mice, BUN and creatinine levels were markedly increased in the response to the presence of the parasite, which reached significant values firstly on day 4 after infection (Figures 1(b) and 1(c)). The infected mice died within 2 weeks (Figure 1(d)).

### 3.2. Protective Effect of Neem Leaf Extract on Renal Damage Induced by* Plasmodium berghei* Infection in Mice

**Figure 2:**
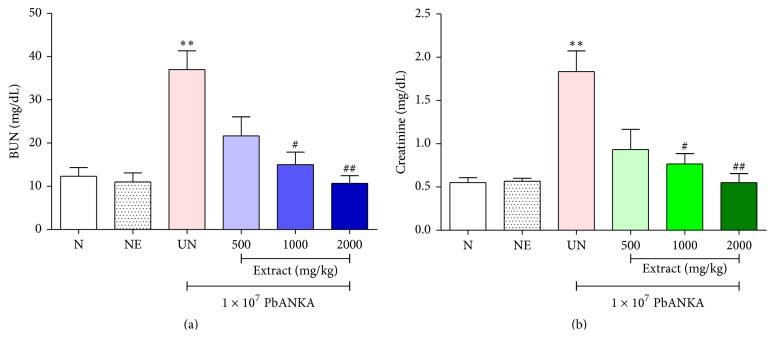
Protective effect of aqueous crude extract of neem leaves on renal injury induced by* Plasmodium berghei* infection in mice. Groups of ICR mice (5 mice of each) were infected with 1 × 10^7^ parasitized erythrocytes of PbANKA by IP injection and given 500, 1,000, and 2,000 mg/kg of neem leaf extracts orally for 4 consecutive days. On day 5 of experiment, (a) BUN and (b) creatinine levels were measured and compared to normal and untreated groups. Results were expressed as mean ± SEM. ^*∗∗*^ *P* < 0.01, compared to normal group, ^#^ *P* < 0.05 and ^##^ *P* < 0.01, compared to untreated group. N: normal mice, NE: normal mice treated with 2,000 mg/kg of neem leaf extract, and UN: untreated mice.

ICR mice were infected with 1 × 10^7^ parasitized erythrocytes of PbANKA by IP injection and given 500, 1,000, and 2,000 mg/kg of the neem leaf extract for 4 consecutive days subsequently with measurement of renal markers. As showed in Figures 2(a) and 2(b), during PbANKA infection, renal damage was developed as indicated by increasing of BUN and creatinine levels significantly (*P* < 0.01). Interestingly, neem leas extract exerted a dose-dependent protection of renal damage induced by PbANKA infection as indicated by reduction of BUN and creatinine levels in the extract treated groups. Significant (*P* < 0.05) protections were observed at doses of 1,000 and 2,000 mg/kg of neem leaf extracts, compared with untreated groups, and no significances were found when compared to normal groups. Moreover, prolonged survival time was also observed in infected mice treated with 1,000 and 2,000 mg/kg of the extracts (25 days for 1,000 mg/kg and 30 days for 2,000 mg/kg). Additionally, no toxic effects were found in normal mice treated with 2,000 mg/kg of this extract.

## 4. Discussion

In the current study, we provide evidence that malaria-associated renal injury could be protected against by using the aqueous crude extract of neem leaves in PbANKA infected mouse model. Impairment of renal function during malaria infection has been reported by clinical reports, and it is an important life-threatening complication of malaria infection that goes beyond the classical clinical symptoms of malaria [22, 23]. Moreover, the adversities to access of medical services or delay in diagnosis in their place of origin is implicated in the severity of disease. The onset of renal injury in PbANKA infected mice came out from day 4 after infection and the incidence of renal injury was confirmed through manifestation including marked increases of plasma BUN and creatinine levels. It can be suggested that pathophysiology of malaria infection was usually associated with an activation of immune system and comprehends a complex network with production of ROS, oxidative stress, and inflammation [24–28]. It has also been described that the erythrocyte destruction during blood stage of infection accumulated high levels of toxic free heme in circulation that had the ability to induce oxidative stress from production of hydroxyl radicals via the Fenton/Haber-Weiss reaction and resulting renal injury [29–31]. Apoptosis of renal tissues during malaria infection has also been suggested in renal injury [30]. Additionally, pathogenesis of malaria-associated renal injury is most likely to be due to immune-complex-mediated glomerulonephritis caused by immune-complex deposition and endothelial damage, which may lead to fatal forms of malarial nephropathies [32].

The present study here has demonstrated that aqueous crude extract of neem leaves significantly reduced plasma BUN and creatinine levels as indicators for renal injury induced by PbANKA infection. This protective effect of neem leaf extract on renal injury may be due to its unique composition, where the leaf extract neem is rich in flavonoids (rutin and quercetin, flavonoglycosides, polyphenols, and tannins) [13]. Flavonoids in neem have been described to possess both antioxidant and anti-inflammatory activities via scavenging free radicals and inhibit lipid peroxidation [33]. Furthermore, neem leaves are rich in polyphenols, which are known for their potent antioxidant and free radical scavenging properties [9, 11]. Therefore, antioxidant and free radical scavenging activities of neem leaf extract might play a critical role to protect against renal injury induced by PbANKA infection in mice. In addition, it has been reported that neem leaf extract in both crude extract and active ingredient, azadirachtin, showed the antimalarial property against PbANKA infected mice and might be due to the effect of this plant to protect against renal injury induced by malaria [34].

## 5. Conclusion

The results obtained in this study showed the aqueous crude extract of neem (*A. indica*) leaves exerted a dose-dependent protective activity of renal damage induced by* P. berghei*. It was most effective at the dose levels of 1,000 and 2,000 mg/kg. This plant can be recommended for use since it possessed a high protective effect against malaria and can be obtained at relatively no cost from nature.
